# Supplementary material for: Maturation of the Visceral (Gut-Adipose-Liver) Network in Response to the Weaning Reaction versus Adult Age and Impact of Maternal High-Fat Diet
Source: Nutrients. 2021 Sep 28;13(10):3438. doi: 10.3390/nu13103438 (PMC8541006; doi:10.3390/nu13103438)
Supplement: Supplementary file 1 [file nutrients-13-03438-s001.zip › nutrients-1360741 supplementary update.pdf]

SUPPLEMENTAL MATERIALS

Table S1. Regression coefficients for associations given in Figure 3AB

Age related associations in pooled groups	Gut-CT caecum	Gut-CT colon	L-CT caecum	L-CT colon	VF-CT caecum	VF-CT colon	Gut-GE10 caecum	Gut-GE10 colon	Gut-GE60 caecum	Gut-GE60 colon	Gut-GU10 caecum	Gut-GU10 colon	Gut-GU60 caecum	Gut-GU60 colon	Steatosi Macro caecum	Steatosi Macro colon	Liver lobular inflamm. caecum	Liver lobular inflamm. colon	Portal inflamm. caecum	Portal inflamm. colon	Score tot colon (ns caecum)	L-GE10 caecum	L-GE10 colon	L-GU10 caecum (ns colon)	VF-GE10 caecum (ns colon)	VF-GE60 caecum (ns colon)	VF-GU10 caecum (ns colon)	VF-GU60 caecum	VF-GU60 colon
g_Adlercreutzia	0,5		0,6	0,5			-0,4	-0,4	-0,5		-0,4	-0,4	-0,4									-0,5		-0,4					
g_Akkermansia						0,5	-0,4				-0,3											-0,5	-0,4		-0,6		-0,5		
g_Bacillus	0,4	0,4		0,4						-0,4				-0,4					-0,5			-0,5							
g_Bifidobacterium			0,5	0,4																									
g_Bilophila		0,4		0,5																									
g_Blautia																0,6				-0,5									
g_Burkholeriaceae_unlc	0,4		0,4		0,4																								
g_Clostridiales_unlc	-0,5			-0,4																		0,3							
g_Coprobacillus							-0,3											0,6											
g_Coprococcus	-0,5						0,4										0,5					0,4							
g_Corynebacterium				-0,4																	0,5								
g_Dehalobacterium	-0,5				-0,4																	0,4							
g_Desulfovibrio	0,4		0,5						-0,4													-0,5	-0,4			-0,4			
g_Lactobacillus	0,7	0,4	0,5																			-0,4							
g_Planococaceae_uncl	0,4																												
g_Oscillospira	-0,5						0,5		0,4		0,4											0,4			0,4	0,3			
g_rc44	-0,6	-0,4	-0,5	-0,4	-0,4	-0,4	0,4		0,3		0,4											0,5	0,5		0,3				
g_RF39_unlc	0,4	0,4	0,4	0,4					-0,4		-0,3		-0,5				0,6									-0,4		-0,4	
g_Rikenellaceae_uncl			0,4																				-0,4						
g_Roseburia	-0,4	-0,5	-0,6	-0,6	-0,4	-0,5																	0,3						
g_Ruminococcaceae_unlc	-0,6	-0,4	-0,4		-0,4	-0,4	0,4				0,3											0,4	0,4						
g_S247_unlc	-0,7	-0,4	-0,5		-0,4		0,4		0,4													0,4		0,3					
g_Streptococcus			0,3																										-0,4
Adipocytokine.signaling.pathway	-0,7						0,3		0,4													0,4							
Arginine.and.proline.metabolism	-0,7	-0,4							0,4													0,4							
D.Alanine.metabolism	0,6	0,5		0,4					-0,3				-0,3																
Energy.metabolism	-0,7								0,3		0,3														0,4		0,4		
Glycine.serine.and.threonine.metabolism	-0,8	-0,4	-0,4						0,4				0,4									0,4							
Glycolysis..Gluconeogenesis	0,7																					-0,3			-0,4		-0,3		
Glycosaminoglycan.degradation	-0,7		-0,4										0,3									0,4		0,4					
Glycosphingolipid.biosynthesis..ganglio.series	-0,7		-0,4				0,4		0,4		0,3											0,5		0,4					
Glycosphingolipid.biosynthesis..globo.series	-0,7	-0,4	-0,4		-0,3	-0,4	0,4		0,4		0,4											0,5		0,4					
Lipoic.acid.metabolism	-0,6												0,3									0,3		0,3					
Lipopolysaccharide.biosynthesis	-0,6																												
Lipopolysaccharide.biosynthesis.proteins	-0,6																							0,3					
Lysine.biosynthesis	-0,5																								0,4	0,3	0,4		
MAPK.signaling.pathway..yeast	-0,8						0,4		0,4		0,4											0,4							
N.Glycan.biosynthesis	-0,7						0,4		0,4		0,4		0,5									0,4		0,5					
Nitrogen.metabolism (also 6 mo)																		-0,6											
Other.glycan.degradation	-0,7		-0,4		-0,4		0,4		0,3		0,3											0,4		0,3					
Oxidative.phosphorylation	-0,6												0,4			-0,6									0,4	0,3	0,4	0,5	
Pentose.phosphate.pathway	0,5																												
Peptidoglycan.biosynthesis	0,6	0,4																											
Peroxisome	-0,8		-0,4				0,4		0,4		0,4		0,4									0,5			0,3				
Phenylalanine.tyrosine.and.tryptophan.biosynthesis	-0,7	-0,4	-0,3																										
Phosphotransferase.system.PTS.	0,6																												
Primary.bile.acid.biosynthesis			-0,5		-0,5	-0,4									0,5	0,5					0,6								
Propanoate.metabolism	0,6						-0,3																						
Secondary.bile.acid.biosynthesis			-0,5		-0,5											0,7													
Sphingolipid.metabolism	-0,7		-0,4				0,4		0,4		0,4											0,4		0,4					
Starch.and.sucrose.metabolism (also 6 mo)	-0,4		-0,5		-0,4	-0,4																							
Sulfur.relay.system	0,6				0,4	0,4	-0,4		-0,4		-0,4											-0,5		-0,4					
Tyrosine.metabolism	0,7		0,3		0,4																								
Valine.leucine.and.isoleucine.biosynthesis	-0,7						0,3		0,4		0,3											0,3					0,3		

Table S2. Regression coefficients for associations given in Figure 3C-F

1 month old offspring	L-CT caecum	L-CT colon	Steatosi Macro caecum	Portal inflamm. caecum	Liver lob. inflamm./score colon	Gut-GU60 colon	VF-GE10 colon	VF-GE60 colon	VF-GU10 colon	VF-GU60 caecum	VF-GU60 colon
f_Peptostreptococcaceae									0,5		0,5
f_Porphyromonadaceae								0,5	0,5		0,6
f_RF39_uncl						0,7					0,6
g_Anaerotruncus						-0,7				-0,5	-0,7
g_Clostridium						0,5					
g_Parabacteroides								0,5	0,5		0,6
g_Peptostreptococcaceae											0,5
g_RF39_uncl						0,7				0,6	0,6
g_Rikenellaceae_uncl							0,6	0,6	0,6		0,6
Butanoate.metabolism				-0,6							
Lysine.degradation	0,5	0,5									
Phenylalanine.metabolism			-0,7		-0,7						0,5
PPAR.signaling.pathway					-0,7						
Pyruvate.metabolism				-0,7							
Tryptophan.metabolism	0,5										

6 months old offspring	Liver lob. inflamm./score caecum	Liver lob. inflamm./score colon	L-GU60 caecum	L-GE60 colon	L-GU60 colon	Gut-GE60 caecum	Gut-GE60 colon	Gut-GU60 caecum	Gut-GU60 colon
f_Clostridiales_uncl	0,8				-0,5			-0,6	
f_Coriobacteriaceae			0,6		0,5				
f_Peptococcaceae					-0,5			-0,5	
f_Turicibacteriaceae							-0,5	-0,6	-0,6
g_Allobaculum					0,6				
g_Clostridiales_uncl	0,8				-0,5			-0,6	
g_Desulfovibrio			0,5						
g_rc44					-0,5			-0,5	
g_Ruminococcaceae_uncl					-0,6				
g_Turicibacter							-0,5	0,6	-0,6
Arachidonic.acid.metabolism						0,5	0,5		0,6
Butanoate.metabolism		-0,8							
Cyanoamino.acid.metabolism	0,9				-0,6				
Cysteine.and.methionine.metabolism				0,5				0,6	
Glycan.biosynthesis.and.metabolism				0,6					
Insulin.signaling.pathway	0,5	0,8							
Methane.metabolism	0,8				-0,6				
Nitrogen.metabolism	-0,8			0,6					
Phenylalanine.metabolism									0,6
Phosphatidylinositol.signaling.system	-0,8		0,5						
Sulfur.metabolism							0,6	0,6	0,6
Taurine.and.hypotaurine.metabolism			0,7						

Figure S1

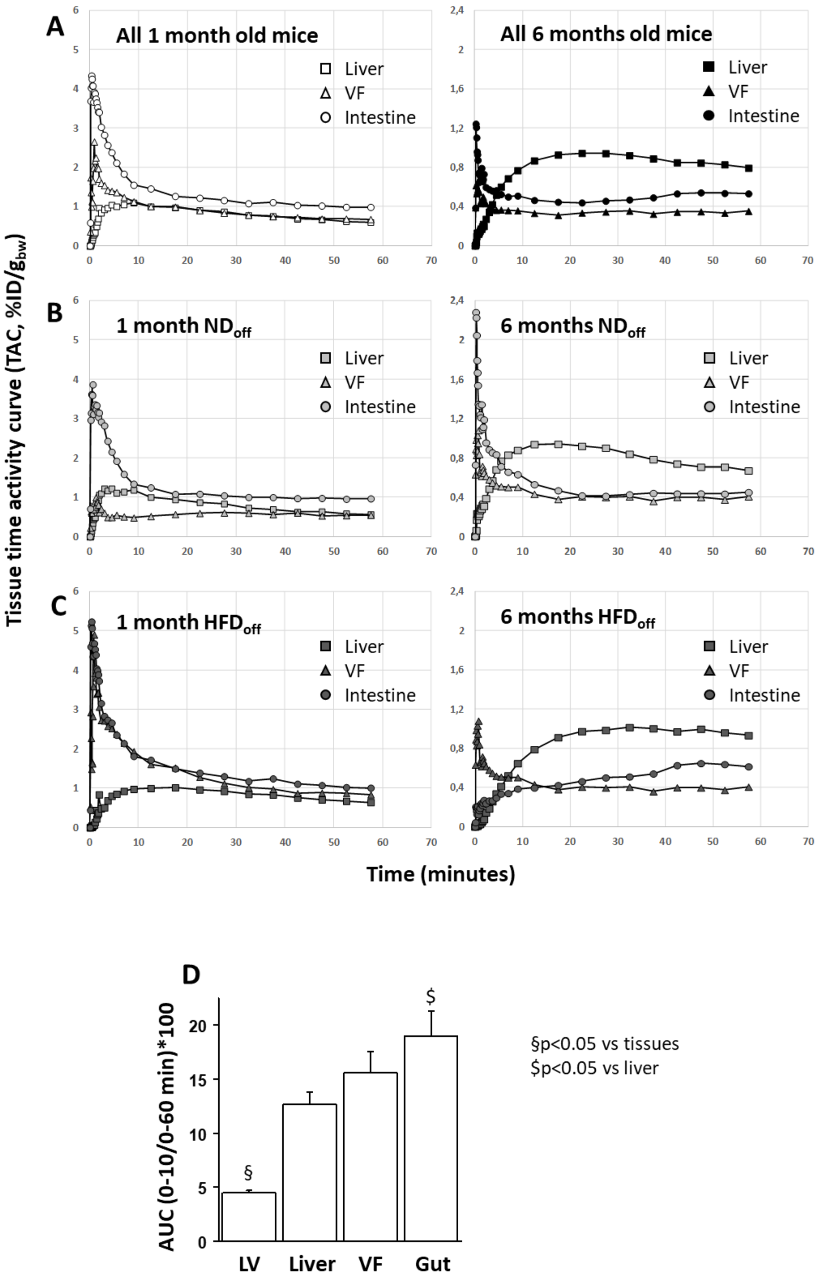

Figure S1. **Radiotracer distribution between tissues.** Top panels depict the time-course and relative proportions of ^{18}FDG reaching the visceral organs, in weaning vs adult mice (A), and in maternal diet*age groups (B-C). Panel D shows the relative amount of FDG reaching arterial blood (left ventricular cardiac cavity, LV) and target organs in the first 10 min from i.p. injection, defining the pre-systemic phase, in which $^{§}p<0.05$ LV vs all tissues, and $^{\$}p<0.05$ gut vs liver.

Figure S2

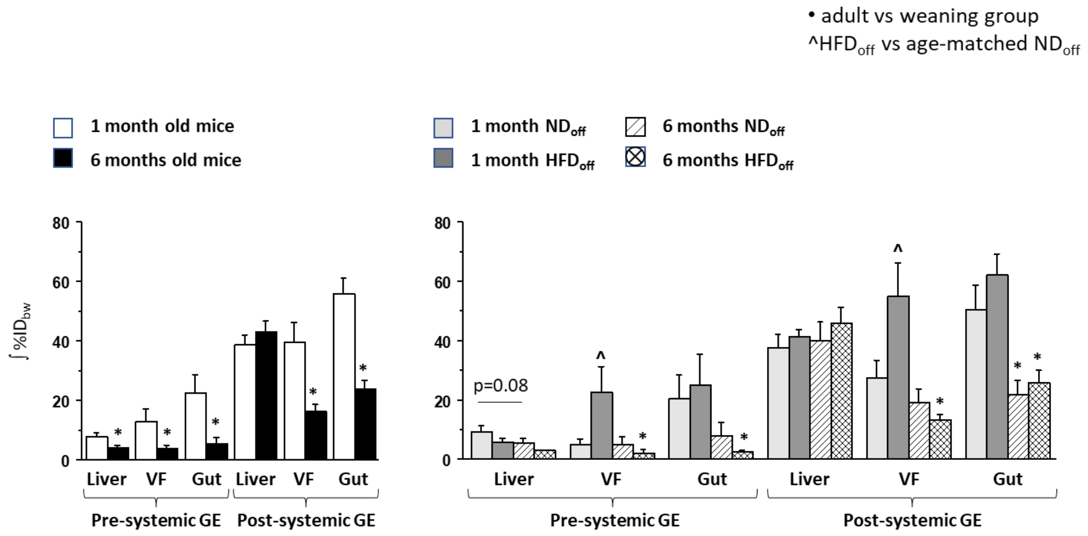

Figure S2: **Glucose fractional extraction.** GE in liver, VF and gut in the offspring, according to age (top panels), or to maternal diet*age (bottom panels). *$p<0.05$ adult vs weaning group, ^$p<0.05$ age-matched HFD_{off} vs NDoff; relevant borderline differences are also shown (text).

Figure S3

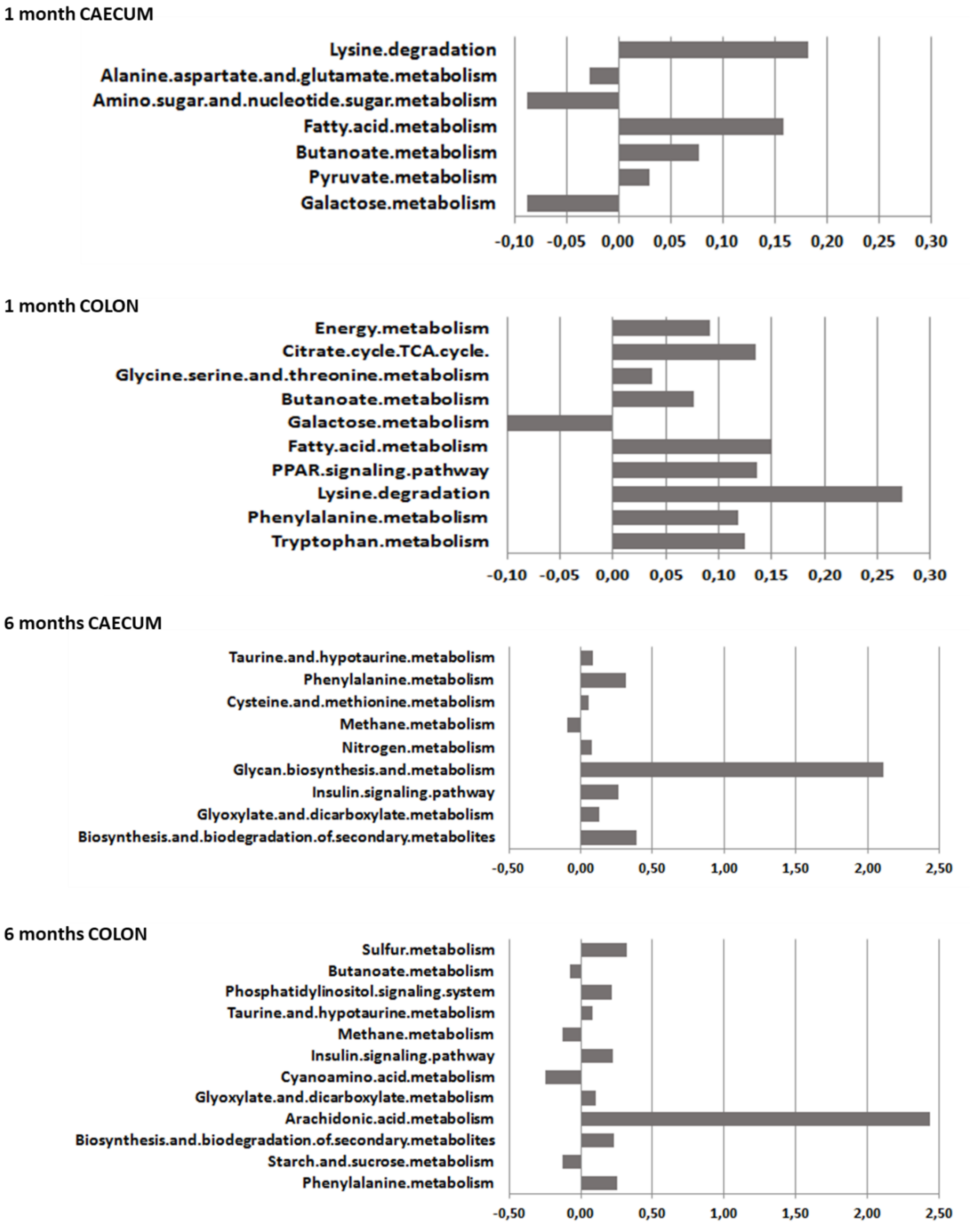

Figure S3: **Colon and caecum KEGG analyses according to maternal diet*age.** Analyses show significantly different metabolic pathways, here expressed as HFD_{off}/ND_{off} ratios in weaning mice (top panels) and adult mice (bottom panels); positive values identify dominant pathways in HFD_{off}, whereas negative values indicate dominance in ND_{off} and deficiency in HFD_{off}.
